# Health outcomes of victim-survivors accessing specialist domestic abuse services: the role of abuse experience, vulnerabilities and sociodemographic characteristics

**DOI:** 10.1186/s12905-026-04518-8

**Published:** 2026-05-23

**Authors:** Annie Bunce, Elouise Davies, Estela Capelas Barbosa

**Affiliations:** 1 Violence and Society Centre, School of Policy and Global Affairs, Rhind Building, City St. George’s, University of London, London, EC1V 0HB UK; 2 Population Health Sciences, Bristol Medical School, University of Bristol, Bristol, UK

**Keywords:** Domestic abuse, health, specialist services, help-seeking, vulnerability, mental health

## Abstract

**Background:**

Domestic abuse (DA) is a public health problem with wide-ranging impacts for victim-survivors and services responding to it. The purpose of the current study was to explore relationships between victim-survivors’ experiences of abuse, additional needs/vulnerabilities and sociodemographic characteristics, and physical and mental health outcomes and health care help-seeking behaviours following DA.

**Methods:**

Secondary analysis was conducted using Women’s Aid Federation of England’s (Women’s Aid) case management and outcomes measurement system, On Track, the largest national dataset on DA. To understand the relationship between abuse types (physical, sexual, emotional, financial, coercive control, technology-facilitated abuse (tech abuse) and threats to kill), needs/vulnerabilities (disability; offending, drug and alcohol-related support needs; pregnancy, recourse to public funds and accessing by-and-for services) and health outcomes (perpetrator caused harm to or loss of unborn child, attempted strangulation, self-harm (disclosed), feeling depressed or suicidal, injury requiring GP treatment and injury requiring Accident and Emergency (A&E) treatment), we used a series of logistic regression models, controlling for potentially confounding variables (including accommodation status, sexual orientation and ethnicity). Stakeholders from Women’s Aid and five other third sector organisations input into the study design and interpretation of results.

**Results:**

Ninety-six percent of victim-survivors accessing DA services (*n =* 77,785) were female. Almost half (41.24%) had felt depressed/suicidal, while 5.59% disclosed having self-harmed. Almost one quarter (23.41%) had suffered a strangulation attempt, and 2.54% had suffered harm to or loss of their unborn child caused by the perpetrator. Just under 10% had an injury requiring A&E treatment and slightly less (7.33%) had an injury requiring GP treatment. Associations with the type of abuse varied by health outcome, for example physical abuse followed by threats to kill were most strongly associated with attempted strangulation and injuries requiring GP and A&E treatment, whilst tech abuse was most strongly associated with self-harm and feeling depressed/suicidal. Vulnerabilities/needs were more consistently associated with health outcomes, with those with a disability; drug, alcohol or offending support needs, or living in temporary/unstable accommodation more likely to experience negative outcomes across the board, and almost all needs/vulnerabilities being associated with adverse mental health outcomes (with the exception of pregnancy and accessing specialist by-and-for services, which appeared to have a protective effect).

**Conclusions:**

Our findings highlight the almost inevitable harms to mental health for victim-survivors of DA, the dangers of non-physical types of abuse such as threats to kill and tech abuse and the heightened risk of attempted strangulation. These findings have particularly important and timely implications for the training of health care professionals. Alongside improvements in health care settings, health care professionals, specialist support workers, researchers and policymakers must continue to explore more integrative and collaborative ways of working to further improve the response to DA and intervene before irreversible damage is done.

## Background

Domestic abuse (DA) is a public health problem [1, 2]. The impacts of DA are wide-ranging, including harms to physical and mental health and, more generally, impacts on victim-survivors’ use of health services [3–6]. These impacts on women’s health often persist long after the violence has stopped and can be fatal [7, 8]. While the current article refers primarily throughout to domestic abuse (DA), a broad term that can include domestic and/or sexual violence and abuse perpetrated by an (ex)-intimate partner or relative, the majority of domestic abuse is perpetrated by (ex)-intimate partners and so much of the literature cited throughout refers to intimate partner violence (IPV). Domestic abuse is the most appropriate term for the current paper as it is the one Women’s Aid use, but it is important to note that 87% of victim-survivors included in our analysis were abused by an (ex)-intimate partner. Where the terms domestic violence (DV), domestic violence and abuse (DVA) and IPV are used instead of DA, this is because we have used the term defined and used by the authors when referring to specific studies.

Research has shown that recent, prolonged and more severe forms of IPV (particularly physical and sexual) are associated with worse physical, mental, and social functioning among women [9], a finding supported by systematic reviews linking IPV to a broad spectrum of adverse health outcomes [10, 11].

### Impact of domestic abuse on physical health

The physical health impacts of DA can be immediate (for example bruises, broken bones or abrasions) or chronic, with the victim-survivor being left with serious injuries that can result in disabilities or long-term health problems. Women are more likely than men to experience physical violence by intimate partners [12] or other domestic relations [13, 14], and have a higher risk of physical injuries [15, 16]. Global estimates attribute a measurable burden of deaths and disability adjusted life years (DALYs) to IPV [17], while UN data highlight the scale of femicide worldwide [18]. Globally, women who have been physically or sexually abused by their partner report higher rates of a number of physical health problems, including having a low birth-weight baby or abortion and acquiring HIV [19]. A landmark US study US study found that women with a history of physical or sexual IPV had significantly higher rates of chronic health problems, including gastrointestinal disorders, chronic pain, and gynaecological issues, compared to non-abused women [9]. A UK-based study by Chandan et al. [3] found that women exposed to DA were 31% more likely to develop cardiovascular disease, had a 51% increased risk of developing type 2 diabetes, and a 44% higher risk of all-cause mortality.

A report by the World Health Organisation (WHO) found that women who experience physical or sexual violence by intimate partners are at greater risk of physical injuries, sexually transmitted infections, unintended or unwanted pregnancies, abortion or unsafe abortions, and pregnancy complications, including miscarriages [7]. Additionally, research finds that pregnant women with experience of DA experience other indirect consequences of abuse and higher levels of high-risk behaviours, including an increase of stress-related conditions and alcohol, tobacco and drug use [20, 21]. In a larger and more recent US study, emotional, physical and sexual abuse were associated with delayed or no prenatal care, depression and substance use during pregnancy and having an infant with low birth weight [22]. Physical, sexual, and any IPV were associated with having a preterm birth, and physical IPV was associated with pregnancy-related hypertension. Maternal exposure to IPV has also been linked to physical health outcomes for children younger than five years, including diarrhoeal disease and acute respiratory infection [23].

Non-fatal strangulation (NFS) is a significant health outcome associated with ongoing DA and has received increasing attention over the past two decades [24, 25]. Non-fatal strangulation was introduced as a new offence in England and Wales in 2022 as part of the Domestic Abuse Act (2021) and it consists of both an action and a harm (a form of abuse and a specific harm to the victim-survivor). It occurs when a person intentionally strangles or restricts another’s ability to breathe and is often used to control and intimidate [26], with most incidents of NFS reported to the police being domestic in nature [25]. Non-fatal strangulation is a particularly gendered form of IPV, disproportionately affecting women and carried out predominantly by men on women [27–29]. A systematic review of studies from 9 different countries found that lifetime prevalence of NFS for women ranged from 3.0—9.7%; with prevalence being 5.5% of women with lifetime experience of IPV in the one English study included [30]. A UK study of the prevalence of NFS amongst clients attending a Sexual Assault Referral Centre (SARC) [31] found that in DA cases, one in five cases involved strangulation, compared to one in 16 in non-DA cases. Additionally, a survey commissioned by BBC 5 Live and Savanta ComRes found that 38% of women under 40 had experienced strangulation during sex, with 42% reporting that this was non-consensual and coercive [32]. Oftentimes, strangulation results in no visible injury (in nearly half of cases according to Green [33]), though it results in feelings of fear, intimidation, and immobilisation [27], and sometimes injuries can manifest hours or days after the act [33]. In this paper, we focus on NFS as an outcome rather than an action.

Research shows that victim-survivors of NFS are at an elevated risk of various other health impacts including traumatic brain injuries, respiratory complications and sometimes, delayed fatal outcomes [24]. Strangulation in a domestic relationship has also been found to be associated with neurological impacts such as loss of consciousness, seizures and paralysis, as well as cognitive impacts such as memory loss [29], and long-term mental health outcomes such as post-traumatic stress disorder (PTSD), fear and anxiety [29, 34]. Non-fatal strangulation is viewed as a key marker for the escalation of domestic violence (DV) [35] and is a risk factor for homicide of women [36, 37].

Therefore, not only does DA lead to negative physical health consequences that can impact victim-survivors in the immediate aftermath and the much longer-term, severe physical health outcomes such as NFS can themselves impact on victim-survivors’ mental health.

### Impact of domestic abuse on mental health

The impact of DA on victim-survivors’ health is not limited to their physical health. In relation to health care DA has been termed a “wicked problem”, particularly complex due to the fact that exposure to IPV, whether in adulthood or in childhood, increases the likelihood of developing a range of mental health problems, and those with mental health problems are at greater risk of exposure to IPV [11, 38–40]. These associations appear to occur across the lifespan, are rooted in gender and other intersecting inequalities, and relate to both the onset and course of mental health problems [11]. Reviews have found that those with mental disorders are at particularly high risk of violence from intimate partners [41–43], and that people with experience of DA are at higher risk of a plethora of adverse outcomes related to mental health and suicide [7, 44, 45].

Whilst many studies have focused on physical or sexual violence, it is increasingly recognised that emotional abuse and control may be the aspects of DV most strongly associated with poor health outcomes, particularly for mental health [46–49]. A US study that explored the independent contribution of four different forms of abuse on mental health found that psychological abuse and stalking contributed uniquely to the prediction of PTSD and depression symptoms, even after controlling for the effects of physical violence, injuries, and sexual coercion [46]. More recently, analysis of data from the Adult Psychiatric Morbidity Survey, a nationally representative survey of the population in England, revealed that receiving threatening or obscene messages from a partner, while often occurring alongside sexual and physical violence, has an additional association with common mental disorder, self-harm and suicidal thoughts [50].

Most studies are cross-sectional and test for associations between DA and indicators of poor mental health, meaning causation cannot be formally inferred, although it is often implied. There are a few exceptions. A systematic review and meta-analysis of 35 separate cohort studies by Bacchus et al. [39] found that five studies demonstrated a positive, statistically significant relationship between depressive symptoms and subsequent IPV. Recent IPV (occurring up to and including the last 12 months) was also positively associated with subsequent depressive symptoms in eight studies, and with increased symptoms of subsequent postpartum depression in five studies. A recent longitudinal study of men and women in Rwanda similarly found evidence for the bi-directional relationship between depression and IPV [51]. A retrospective cohort study of female survivors of IPV drawing on UK primary care records found that just under half of women in the exposed group had some form of mental illness (compared to just under a quarter in the unexposed group), and, when excluding those with mental illness at baseline, women exposed to IPV were over twice as likely to go on to present with any type of mental illness [52].

### Healthcare seeking behaviour in victim-survivors of domestic abuse

Given the strong evidence that experiencing DA impacts both physical and mental health outcomes, victim-survivors often have high need for a plethora of services, including health care [53]. While there is a growing literature on their use of specialist support services, fewer studies have explored healthcare service utilisation by victim-survivors of DA [6]. This is likely in part due to a comparative lack of disclosure in health care settings; a result of both victim-survivors lack of trust in statutory agencies and fears around confidentiality (including the potential misuse of personal information), and health care professionals’ lack of confidence and training to enquire about DA [54, 55].

Much of the existing evidence that has explored healthcare usage of victim-survivors of DA is qualitative. For example, a qualitative study of pathways towards formal and informal support for women seeking help from specialist DVA agencies in the UK identified the role of health professionals as a key theme [54]. The study found that, although twenty-seven of the thirty-one women interviewed had consulted their GP for help with anxious and depressed feelings, none of them had the intention of talking to their GP about DA. For those who did eventually disclose abuse to their GP, contacting the specialist DA service had legitimised further disclosure and triggered better support from the GP, although ultimately overall the GP response was considered unsatisfactory and disclosing abuse a waste of time. These findings mirror those from another qualitative study conducted over a decade earlier, which specifically explored experiences of seeking help from health professionals in a sub-sample of women in the postpartum period who experienced DV [55]. This study found that women scored highly on measures of postnatal depression and PTSD and regarded GPs and A&E staff as less helpful compared with health visitors in responding to DV. Very few women voluntarily disclosed DV to a health professional and fewer still were asked directly about DV by one.

Together these findings highlight that victim-survivors of DA have a high need for health care services. However, negative experiences of disclosing DA to health care professionals may put them off approaching them for help in the future, and providing adequate support for DA in health care settings is an ongoing challenge. In UK general practice, there is evidence from mixed methods studies on how the national IRIS (Identification and Referral to Improve Safety) model, a programme of training and support in primary health care practices to increase identification of women experiencing domestic violence and abuse (DVA) and their referral to specialist advocacy services, is improving health care responses to women affected by DA [56–58]. There is also emerging evidence for the potential of the adapted IRIS + model for identifying and responding to men and children and young people (CYP) affected by DA, although additional barriers persist when it comes to identifying and supporting CYP and male perpetrators of DA [59, 60].

A systematic review exploring how DA impacts upon disabled women’s access to maternity services [61] included eleven studies in total, three of which concluded that DA is significantly associated with delayed entry into antenatal care for women with and without a mental health condition, suggesting that DA and mental conditions have independent effects on service access and utilisation [62–64]. Where studies focused on women with physical and sensory impairments, however, the effects of DA were found to compound existing barriers to their access and utilisation of maternity care. Although the review identified DA as a barrier to accessing services, three of the included studies highlighted that the health consequences of DA can prompt women to access services more quickly or utilise services more frequently [21, 64, 65]. Research finds that pregnant women with experience of DA are admitted to hospital more frequently during their pregnancies than non-abused pregnant women [20]. Women hospitalized during pregnancy, particularly those with substance abuse and mental health-related conditions, have been found to be at high risk for concurrent IPV, especially physical (as opposed to nonphysical, or any) IPV [66]. Another study by Huth-Bocks et al. [64] found that women experiencing both physical and emotional abuse had longer hospital stays, more frequent visits to the emergency room and a higher number of visits to their doctor for the infant during the postnatal period. Additionally, a longitudinal cohort study in the US found not only that women with a history of IPV had significantly higher healthcare utilization and costs which continued long after IPV ended [67], but also that children of mothers with a history of IPV that ended before the child was born had significantly greater utilization of mental health, primary care, specialty care, and pharmacy services [68], highlighting the prolonged impact of DA.

While existing literature demonstrates that experience of DA- and its physical and mental health consequences- can both compromise, and catalyse, access to services, this has seldom been explored using data on health and healthcare collected by specialist DA support services. The current study aimed to build on the existing evidence base by drawing upon a large national administrative dataset of DA services to explore quantitatively associations between various factors and both physical and mental health outcomes, and health care seeking behaviour.

### Aims and research questions

The primary aim of this study was to explore relationships between victim-survivors’ needs, vulnerabilities and other characteristics, and their health outcomes following domestic abuse. To achieve this aim, we explored the following research questions:

1. Which factors are associated with physical health outcomes (attempted strangulation and harm to or loss of unborn child) of victim-survivors accessing specialist domestic abuse support services?
2. Which factors are associated with mental health outcomes (self-harm and feeling depressed or suicidal) of victim-survivors accessing specialist domestic abuse support services?
3. Which factors are associated with healthcare seeking behaviour (injury requiring GP treatment and injury requiring A&E treatment) of victim-survivors accessing specialist domestic abuse support services?

## Methods

### Data description

Data for this study was provided by Women’s Aid, a federation of over 170 DA services which provide around 300 local services to women and children across England [69]. Their work aims to end abuse against women and children, via the provision of frontline services for victim-survivors ranging from children’s services to financial advice, mental health support, employment advice and support with finding safe housing or accommodation. Women’s Aid’s support services include a Live Chat Helpline, the Survivors’ Forum, the No Woman Turned Away Project, a dedicated website for young people in their first relationship (Love Respect), and advocacy projects (among others) [69].

Specifically, this paper analysed data from On Track, Women’s Aid’s case management and outcomes measurement system and the largest national dataset on DA in England. The system is used day-to-day by frontline staff to record information about service users and to measure outcomes of the service [70]. Every region in England is represented within On Track, with over 100 local DA services now using the system (not all of them Women’s Aid member services). The anonymised dataset collects information on the sociodemographics of the victim-survivor and alleged perpetrator(s), experiences of abuse of victim-survivors accessing the services, and their support needs, outcomes, and referral patterns. There is also feedback and information about experiences of external services such as housing, legal services, NHS and police. On Track was launched in April 2016, and data for this study included data collected between 1 st April 2016 and 31 st March 2023.

The data files were provided to the researchers following the removal of all personal identifiers. The raw data was transferred to researchers in separate Excel files which contained information on: demographics; alleged perpetrators; adult service records; the nature of the abuse; support needs identified; referrals made, and service exits. These anonymised Excel files were uploaded securely by the researchers into a Trusted Research Environment (TRE) and then exported into Stata 17 to conduct all data management and statistical analysis. Firstly, data cleaning was carried out on each separate file, which mainly required recoding string variables into binary or categorical variables to enable statistical analysis. Once all data were cleaned, any variables not needed for the planned analysis were dropped from each individual Stata file to make the process of merging the files together more manageable. The current paper draws on data from two of the datasets: adult cases (which contains demographic information and information relating to victim-survivors’ support needs) and adult abuse (which contains information about the nature of the abuse experienced). The adult cases data file included a unique identifier for each victim-survivor (case ID), and information recorded in the adult abuse file was linked to the victim-survivor using this case ID, meaning the case ID variable could be used to merge the two data files together in Stata. As the case ID was only unique in the adult cases file (that is, in the adult abuse data there could be multiple rows with the same case ID), the files could not be merged on a one-to-one basis so instead were merged on a one-to-many basis. We relied on information from Women’s Aid staff that On Track is a sequential SQL-based dataset and thus, information exported have a chronological/ordinal nature. The final merged dataset contained 188,986 observations.

As is common when working with administrative data, there was significant missing data on most variables, for example accommodation type was 32% missing, and whether the service user lived with the perpetrator was 35% missing. Missing data was dealt with using listwise deletion, meaning only cases with complete data on all the variables of interest were included in analyses. Approximately 55% of the initial dataset was excluded for this reason, resulting in 77,785 cases being included in the models. While this approach reduced our sample size, we opted against using multiple imputation or other data replacement techniques because the data were not missing at random. Exploratory analyses indicated that those with missing data had different health or health services outcomes, suggesting systematic differences from the analytic sample. As such, imputing values would have introduced unjustified assumptions about the similarity between those with and without missing data, potentially biasing results. Children (under 16) were included in the analysis as they made up a very small minority (0.02%, *n =* 12) of cases. We conducted a complete case analysis, excluding observations with missing data on any of the primary variables included in our models.

### Measurement

All coding of the categorical variables for analysis was done by two researchers and reviewed by a third. Decisions related to the coding/categorisation of variables were also discussed with stakeholders from Women’s Aid, to ensure the researchers were interpreting the data correctly.

#### Health outcomes of domestic abuse

This paper focuses on which factors are associated with health outcomes of victim-survivors accessing specialist DA services and their health care seeking behaviour. We examined six binary (yes/no) outcome variables which can be grouped into three concepts: physical health outcomes, mental health outcomes and accessing health services.

The physical health outcomes included in the data are attempted (non-fatal) strangulation and perpetrator caused harm to or loss of unborn child (hereon also referred to as ‘harm to or loss to unborn child’),^1^ both of which are significant physical impacts of DA. This paper considers two mental health outcomes: self-harm and feeling depressed/suicidal, which are similarly well-established impacts of DA. Finally, accessing health services was measured using two variables: whether the abuse resulted in injury requiring 1) GP treatment; or 2) A&E treatment.

Victim-survivors can disclose multiple health outcomes, can access DA services multiple times and may disclose different abuse experiences and/or different health outcomes on different occasions. The current analysis was conducted at the case level, meaning some victim-survivors will appear in the data multiple times.

#### Determinants of health outcomes of domestic abuse

We identified three key determinants of the six health outcomes to explore in this study: abuse type, support needs and vulnerabilities. See Table 1 for how these variables were categorised in the models. Due to high levels of missingness on more specific need variables, this study uses three broad categories of need: offending; drug and alcohol-related, selected because of their possible influence on help-seeking behaviour and impact on health. These support need variables are not mutually exclusive, but rather a range of binary variables, meaning victim-survivors could have one, more or any combination of support needs.

**Table 1:** Descriptive Statistics of cases with complete data recorded in Women’s Aid’s On Track system from 1 st April 2016- 31 st March 2023

**Variable**	Total sample (*N* = 77,785)	Attempted strangulation *N* = 18,210 (23.41%)		Harm to or loss of unborn child *N* = 1,975 (2.54%)		Self-harm *N* = 4,347 (5.59%)		Feeling depressed/suicidal *N* = 32,078 (41.24%)		Injury requiring GP treatment *N* = 5,702 (7.33%)		Injury requiring A&E treatment *N* = 7.259 (9.32%)	
	**N (%)**	**N (%)**	*p*-value	**N (%)**	*p*-value	**N (%)**	*p*-value	**N (%)**	*p*-value	**N (%)**	*p*-value	**N (%)**	*p*-value
Type of Abuse													
Emotional	65,396 (84.07)	16,502 (90.62)	<.001	1,798 (91.04)	<.001	4,039 (92.91)	<.001	29,448 (91.80)	<.001	5,073 (88.97)	<.001	6,357 (87.69)	<.001
Physical	44,008 (56.57)	16,044 (88.11)	<.001	1,615 (81.77)	<.001	3,192 (73.43)	<.001	20,851 (65.00)	<.001	5,025 (88.13)	<.001	6,548 (90.33)	<.001
Sexual	15,528 (19.96)	5,950 (32.67)	<.001	800 (40.51)	<.001	1,782 (40.99)	<.001	9,153 (28.53)	<.001	1,899 (33.13)	<.001	2,263 (31.22)	<.001
Financial	28,012 (36.01)	8,386 (46.05)	<.001	1,030 (52.15)	<.001	2,298 (52.86)	<.001	15,108 (47.10)	<.001	2,943 (51.61)	<.001	3,294 (45.44)	<.001
Coercive control	49,741 (63.95)	14,405 (79.10)	<.001	1,560 (78.99)	<.001	3,424 (78.77)	<.001	23,878 (74.44)	<.001	4,279 (75.04)	<.001	5,315 (73.32)	<.001
Technology facilitated	9,335 (12.00)	3,719 (20.42)	<.001	603 (30.53)	<.001	1,225 (28.18)	<.001	5,822 (18.15)	<.001	1,475 (25.87)	<.001	1,613 (22.25)	<.001
Threats to kill	21,974 (28.25)	11,068 (60.78)	<.001	1,145 (57.97)	<.001	1,990 (45.78)	<.001	12,333 (38.45)	<.001	3,331 (58.42)	<.001	4,024 (55.51)	<.001
Vulnerabilities/needs													
Disability													
Yes	25,554 (32.86)	6,238 (34.26)	<.001	713 (36.10)	0.002	2,391 (55.00)	<.001	13,146 (41.04)	<.001	2,118 (37.14)	<.001	2,991 (41.26)	<.001
No	52,231 (67.14)	11,972 (65.74)		1,262 (63.90)		1,956 (45.00)		18,914 (58.96)		3,584 (62.86)		4,258 (58.74)	
Offending support need	2,165 (2.79)	707 (3.88)	<.001	95 (4.81)	<.001	307 (7.06)	<.001	1,034 (3.22)	<.001	298 (5.23)	<.001	437 (6.03)	<.001
Drug support need	3,534 (4.54)	1,136 (6.24)	<.001	127 (6.43)	<.001	588 (13.53)	<.001	1,856 (5.79)	<.001	445 (7.80)	<.001	649 (8.95)	<.001
Alcohol support need	4,227 (5.43)	1,283 (7.05)	<.001	114 (5.77)	0.502	657 (15.11)	<.001	2,171 (6.77)	<.001	540 (9.47)	<.001	824 (11.37)	<.001
Pregnant													
Yes	4,696 (6.04)	1,182 (6.49)	0.003	411 (20.81)	<.001	274 (6.30)	0.448	1,752 (5.46)	<.001	277 (4.86)	<.001	490 (6.76)	0.007
No	73,089 (93.96)	17,028 (93.51)		1,564 (79.19)		4,073 (93.70)		30,326 (94.54)		5,425 (95.14)		6,759 (93.24)	
Recourse to public funds													
Yes	74,769 (96.12)	17,501 (96.11)	0.898	1,862 (94.28)	<.001	4,260 (98.00)	<.001	30,830 (96.11)	0.873	5,424 (95.12)	<.001	7,000 (96.57)	0.040
No	3,016 (3.88)	709 (3.89)		113 (5.72)		87 (2.00)		1,248 (3.89)		278 (4.88)		249 (3.43)	
By-and-for													
Yes	211 (0.27)	31 (0.17)	0.003	< 10	0.876	12 (0.28)	0.950	59 (0.18)	<.001	15 (0.26)	0.902	14 (0.19)	0.179
No	77,574 (99.73)	18,179 (99.83)		1,970 (99.75)		4,335 (99.72)		32,019 (99.82)		5,687 (99.74)		7,235 (99.81)	
Perpetrator living at address													
Perpetrator living at address all of the time	13,977 (17.97)	2,426 (13.32)	<.001	207 (10.48)	<.001	679 (15.62)	<.001	5,652 (17.62)	0.073	779 (13.66)	<.001	991 (13.67)	<.001
Perpetrator living at address some of the time	2,122 (2.73)	480 (2.64)		43 (2.18)		124 (2.85)		860 (2.68)		171 (3.00)		191 (2.63)	
Perpetrator not living at address	61,686 (79.30)	15,304 (84.04)		1,725 (87.34)		3,544 (81.53)		25,566 (79.70)		4,752 (83.34)		6,067 (83.69)	
Sociodemographics													
Accommodation type													
Owner/Occupier	10,508 (13.51)	1,670 (9.17)	<.001	136 (6.89)	<.001	318 (7.32)	<.001	4,032 (12.57)	<.001	591 (10.36)	<.001	631 (8.70)	<.001
Other/temporary	11,796 (15.16)	2,787 (15.30)		331 (16.76)		877 (20.17)		5,055 (15.76)		888 (15.57)		1,149 (15.85)	
In care	131 (0.17)	34 (0.19)		< 10		20 (0.46)		53 (0.17)		17 (0.30)		25 (0.34)	
Healthcare system	6,641 (8.54)	1,343 (7.38)		103 (5.22)		258 (5.94)		2,244 (7.00)		33 (5.84)		520 (7.17)	
Social housing	28,569 (36.73)	7,349 (40.36)		850 (43.04)		1,646 (37.87)		12,212 (38.07)		2,321 (40.71)		2,977 (41.07)	
Military accommodation	45 (0.06)	13 (0.07)		< 10		< 10		16 (0.05)		< 10		< 10	
Private sector	15,150 (19.48)	3,593 (19.73)		338 (17.11)		735 (16.91)		6,173 (19.24)		975 (17.10)		1,285 (17.73)	
Rough sleeper	53 (0.07)	14 (0.08)		< 10		< 10		26 (0.08)		< 10		< 10	
Refuge/shelter	4,770 (6.13)	1,376 (7.56)		211 (10.68)		474 (10.90)		2,211 (6.89)		567 (9.94)		640 (8.83)	
Student accommodation	122 (0.16)	31 (0.17)		< 10		13 0.30		56 (0.17)		< 10		< 10	
Relationship status													
Civil Partnership	276 (0.35)	42 (0.23)	<.001	< 10	<.001	14 (0.32)	<.001	84 (0.26)	<.001	< 10	<.001	16 (0.22)	<.001
Cohabiting	6,150 (7.91)	1,232 (6.77)		144 (7.29)		374 (8,60)		2,574 (8.02)		345 (6.05)		524 (7.23)	
Divorced	1,724(2.22)	337 (1.85)		37 (1.87)		81 (1.86)		713 (2.22)		132 (2.31)		128 (1.77)	
In relationship but not cohabiting	4,716(6.06)	1,123 (6.17)		114 (5.77)		403 (9.27)		1,989 (6.20)		298 (5.23)		465 (6.41)	
Married	12,015 (15.45)	2,181 (11.98)		220 (11.14)		401 (9.22)		4,802 (14.97)		733 (12.86)		779 (10.75)	
Other	601(0.77)	118(0.65)		17 (0.86)		29 (0.67)		218 (0.68)		51 (0.89)		42 (0.58)	
Separated	18,853 (24.24)	4,419 (24.27)		453 (22.94)		784 (18.04)		7,421 (23.13)		1,451 (25.45)		1,700 (23.45)	
Single	33,044 (42.48)	8,703 (47.79)		982 (49.72)		2,252 (51.81)		14,110 (43.99)		2,647 (46.42)		3,545 (48.90)	
Widowed	406(0.52)	55 (0.30)		< 10		< 10		167 (0.52)		37 (0.65)		50 (0.69)	
Region													
East Midlands	14,377 (18.48)	2,909 (15.97)	<.001	287 (14.52)	<.001	643 (14.79)	<.001	5,972 (18.62)	<.001	874 (15.33)	<.001	1,130 (15.59)	<.001
East of England	10,980 (14.12)	2,836 (15.57)		301 (15.24)		699 (16.08)		4,804 (14.98)		814 (14.28)		1,023 (14.11)	
London	7,091 (9.12)	1,847 (10.14)		210 (10.63)		307 (7.06)		2,876 (8.97)		613 (10.75)		719 (9.92)	
Northeast England	3,530 (4.54)	674 (3.70)		48 (2.43)		184 (4.23)		1,015 (3.16)		183 (3.21)		383 (5.28)	
Northwest England	11,483 (14.76)	2,507 (13.77)		312 (15.80)		683 (15.71)		5,245 (16.35)		747 (13.10)		1,085 (14.97)	
Southeast England	13,815 (17.76)	3,670 (20.15)		340 (17.22)		727 (16.72)		5,628 (17.54)		1,062 (18.63)		1,312 (18.10)	
Southwest England	5,396 (6.94)	1,296 (7.12)		148 (7.49)		339 (7.80)		2,661 (8.30)		460 (8.07)		489 (6.75)	
West Midlands	6,670 (8.57)	1,220 (6.70)		164 (8.30)		403 (9.27)		2,094 (6.53)		487 (8.54)		545 (7.52)	
Yorkshire and Humberside	4,443 (5.71)	1.251 (6.87)		165 (8.35)		362 (8.33)		1,783 (5.56)		462 (8.10)		563 (7.77)	
Sexuality													
Heterosexual	75,109 (96.56)	17,528 (96.25)	<.001	1,895 (95.95)	<.001	3,935 (90.52)	<.001	30,642 (95.52)	<.001	5,483 (96.16)	0.021	6,944 (95.79)	<.001
Gay	377 (0.48)	86 (0.47)		< 10		40 (0.92)		202 (0.63)		30 (0.53)		47 (0.65)	
Bisexual	1,514 (1.95)	1,514 (1.95)		67 (3.39)		247 (5.68)		818 (2.55)		136 (2.39)		184 (2.54)	
Asexual	49 (0.06)	< 10		< 10		< 10		23 (0.07)		< 10		< 10	
Lesbian	596 (0.77)	120 (0.66)		< 10		81 (1.86)		308 (0.96)		42 (0.74)		63 (0.87)	
Pansexual	104(0.13)	24 (0.13)		< 10		27 (0.62)		59 (0.18)		< 10		< 10	
Queer	36 (0.05)	< 10		< 10		11 (0.25)		26 (0.08)		< 10		< 10	
Ethnicity													
White	61,022 (78.45)	14,312 (78.59)	<.001	1,437 (72.76)	<.001	3,654 (84.06)	<.001	25,277 (78.80)	0.025	4,378 (76.78)	0.026	5,886 (81.20)	<.001
Asian, Asian British, Asian Welsh	7,332 (9.43)	1,638 (9.00)		237 (12.00)		308 (7.09)		3,020 (9.41)		588 (10.31)		529 (7.30)	
Black, Black British, Black Welsh, Caribbean	5,162 (6.64)	1,140 (6.26)		162 (8.20)		147 (3.38)		2,020 (6.30)		406 (7.12)		446 (6.15)	
Mixed or Multiple ethnic groups	2,654 (3.41)	714 (3.92)		87 (4.41)		172 (3.96)		1,109 (3.46)		199 (3.49)		272 (3.75)	
Other ethnic groups	1,615 (2.08)	406 (2.23)		52 (2.63)		66 (1.52)		652 (2.03)		131 (2.30)		116 (1.60)	
Gender													
Female	74,882 (96.27)	17,881 (98.19)	<.001	1,959 (99.19)	<.001	4,170 (95.93)	<.001	30,771 (95.93)	<.001	5,535 (97.07)	0.003	6,992 (96.45)	0.154
Male	2,834 (3.64)	316 (1.74)		15 (0.76)		161 (3.70)		1,270 (3.96)		165 (2.89)		255 (3.52)	
Queer, intersex, nonbinary, other	69 (0.09)	13 (0.07)		< 10		16 (0.37)		37 (0.12)		< 10		< 10	

The On Track data includes victim-survivors that come into contact with specialist DA services for their experiences of current abuse and historic (lifetime) abuse. Additionally, there were over 20 forms of abuse recorded in the dataset, including: domestic abuse; sexual exploitation; female genital mutilation (FGM); childhood sexual abuse (CSA), stalking and harassment, financial abuse, and more. For this study, the abuse types were categorised into seven groups: physical; sexual; emotional; and financial abuse, and coercive control, to broadly match those reported in Women’s Aid’s annual audits (e.g. 68). We also included two additional categories of tech abuse and threats to kill, due to growing recognition of the prevalence and wide-ranging impacts of online harassment, digital coercion, and cyberstalking [66, 71, 72], and threats to kill as a significant predictor of health harm and needing health care [73–76]. Each of the abuse types were coded as binary yes/no (0,1) variables, whereby multiple abuse types could be recorded for victim-survivors. These abuse types are not mutually exclusive; many victim-survivors may face multiple intertwined abuses—physical, emotional, financial —that collectively compound their risk of harm and need for health services.

While we inherited the categories of abuse used by Women’s Aid, we acknowledge that there are overlaps between various forms of abuse, such as coercive control, threats to kill and emotional abuse. Measuring coercive control, particularly, requires innovative ways of aggregating and categorizing data [77]. Conceptually, these forms of abuse can be seen as on a trajectory of escalation; for example, coercive control might begin earlier (given that it is at the heart of DA] and not yet have escalated into threats to kill (although more longitudinal research is needed here]. Threats of violence and threats to kill can also be conceptually differentiated, although there may be an overlap between them. As highlighted in our background section, threats to kill are also a risk factor for intimate partner and domestic homicide. The degree of distinction and overlap between coercive control and emotional (or psychological] abuse is more complex, but coercion can be differentiated as an attempt by the perpetrator to have overall control of the victim-survivor through the deployment of a credible threat of violence, while not all emotional abuse is controlling even if it is damaging [78].

The third determinant of health outcomes was victim-survivor vulnerabilities. These included whether the perpetrator is living at the address (i.e. whether the victim-survivor is cohabiting or living at the same address as the perpetrator, either all the time or intermittently), whether the victim-survivor has a disability and whether the victim-survivor is pregnant (at the time of accessing support). These also included whether the victim-survivor has recourse to public funds, which indicates whether the victim-survivor is eligible to claim UK state benefits, housing support and tax credits. Some examples of instances where a person may have no recourse to public funds (NRPF) includes those living in the UK on certain visas, illegally or seeking asylum. Additionally, whether the victim-survivor was accessing by-and-for services was also included as a vulnerability. By-and-for services are services run ‘by and for’ the people they serve, for example Black and ethnic minority women or LGBT victim-survivors.

### Statistical methods

Data management, descriptive analyses and regression analyses were conducted in Stata 17. To understand which factors are associated with certain health outcomes for victim-survivors, multivariate logistic regressions were used, controlling for potentially confounding variables. Sociodemographic characteristics of accommodation type, relationship status, region, sexuality, ethnicity, gender and age were controlled for in all models. Regression results are reported as adjusted odds ratios (aORs) and p-values and 95% confidence intervals (CIs) are presented. Too few observations of harm or loss to unborn baby were present for those who were in military accommodation or sleeping rough and those whose sexuality was queer, therefore the coefficients/p-values for these sociodemographics are not presented to avoid misinterpretation. The results can be found in Table 2.

**Table 2:** Logistic regression model results: victim-survivors’ health outcomes based on type of abuse, vulnerabilities/needs and sociodemographic factors

	**Attempted strangulation**		**Harm to or loss of unborn child**		**Self-harm**		**Feeling depressed/suicidal**		**Injury requiring GP treatment**		**Injury requiring A&E treatment**	
	aOR (95% CI)	*P* value	aOR (95% CI)	*P* value	aOR (95% CI)	*P* value	aOR (95% CI)	*P* value	aOR (95% CI)	*P* value	aOR (95% CI)	*P* value
Type of Abuse												
Physical abuse	6.037 (5.725, 6.366)	<.001	2.130 (1.873, 2.422)	<.001	1.281 (1.184, 1.385)	<.001	1.116 (1.079, 1.155)	<.001	4.973 (4.546, 5.441)	<.001	7.368 (6.756, 8.035)	<.001
Sexual abuse	1.433 (1.369, 1.500)	<.001	1.692 (1.530, 1.872)	<.001	1.907 (1.777, 2.0455)	<.001	1.646 (1.583, 1.713)	<.001	1.224 (1.148, 1.306)	<.001	1.205 (1.136, 1.279)	<.001
Emotional abuse	0.628 (0.586, 0.673)	<.001	0.898 (0.749, 1.076)	0.244	1.442 (1.261, 1.650)	<.001	1.802 (1.709, 1.900)	<.001	0.577 (0.521, 0.639)	<.001	0.512 (0.469,.560)	<.001
Financial abuse	1.106 (1.060, 1.154)	<.001	1.383 (1.249, 1.531)	<.001	1.503 (1.401, 1.612)	<.001	1.591 (1.537, 1.645)	<.001	1.324 (1.245, 1.409)	<.001	1.086 (1.027, 1.149)	0.004
Coercive control	1.353 (1.288, 1.422)	<.001	1.064 (0.935, 1.211)	0.348	1.185 (1.085, 1.293)	<.001	1.374 (1.323, 1.426)	<.001	0.952 (0.884, 1.026)	0.198	0.929 (0.870, 0.993)	0.029
Tech abuse	1.531 (1.449, 1.618)	<.001	2.142 (1.924, 2.385)	<.001	2.185 (2.021, 2.363)	<.001	2.102 (2.002, 2.208)	<.001	2.159 (2.012, 2.317)	<.001	1.744 (1.631, 1.865)	<.001
Threats to kill	4.526 (4.350, 4.709)	<.001	2.137 (1.935, 2.361)	<.001	1.415 (1.319, 1.518)	<.001	1.737 (1.676, 1.799)	<.001	2.464 (2.320, 2.616)	<.001	2.322 (2.200, 2.451)	<.001
Vulnerabilities/needs												
Disability	1.022 (0.979, 1.066)	0.329	1.107 (1.002, 1.225)	0.047	2.179 (2.038, 2.330)	<.001	1.805 (1.745, 1.867)	<.001	1.056 (0.993, 1.123)	0.085	1.270 (1.202, 1.342)	<.001
Offending support need	1.213 (1.083, 1.360)	0.001	1.460 (1.156, 1.844)	0.002	1.352 (1.168, 1.565)	<.001	1.036 (0.940, 1.143)	0.475	1.437 (1.246, 1.658)	<.001	1.497 (1.321, 1.696)	<.001
Drug support need	1.043 (0.951, 1.145)	0.369	0.970 (0.786, 1.197)	0.778	1.660 (1.480, 1.863)	<.001	1.201 (1.109, 1.301)	<.001	1.202 (1.064, 1.358)	0.003	1.230 (1.106, 1.368)	<.001
Alcohol support need	1.177 (1.080, 1.282)	<.001	0.992 (0.798, 1.233)	0.944	2.297 (2.060, 2.561)	<.001	1.307 (1.215, 1.405)	<.001	1.385 (1.240, 1.548)	<.001	1.641 (1.492, 1.806)	<.001
Pregnant	0.992 (0.953, 1.033)	0.698	1.930 (1.816, 2.051)	<.001	0.960 (0.898, 1.027)	0.237	0.937 (0.906, 0.969)	<.001	0.902 (0.845, 0.963)	0.002	1.072 (1.017, 1.129)	0.009
Recourse to public funds	1.043 (0.937, 1.160)	0.443	0.883 (0.709, 1.100)	0.269	1.485 (1.178, 1.873)	0.001	0.960 (0.882, 1.046)	0.353	0.771 (0.668, 0.890)	<.001	0.926 (0.799, 1.074)	0.310
By-and-for	0.653 (0.419, 1.017)	0.059	0.905 (0.360, 2.274)	0.831	1.540 (0.834, 2.844)	0.168	0.583 (0.423, 0.805)	0.001	0.957 (0.547, 1.676)	0.879	0.942 (0.531, 1.671)	0.838
Perpetrator living at address all of the time	0.958 (0.901, 1.018)	0.167	0.851 (0.722, 1.002)	0.052	1.436 (1.300, 1.588)	<.001	1.112 (1.062, 1.165)	<.001	0.926 (0.845, 1.014)	0.098	0.964 (0.888, 1.047)	0.388
Perpetrator living at address some of the time	1.027 (0.911, 1.158)	0.660	0.919 (0.671, 1.260)	0.601	1.166 (0.9567, 1.421)	0.128	1.065 (0.968, 1.172)	0.197	1.131 (0.956, 1.339)	0.152	0.901 (0.767, 1.058)	0.203
Sociodemographics												
Accommodation type												
Other/temporary	1.062 (0.978, 1.154)	0.152	1.015 (0.816, 1.262)	0.897	1.762 (1.524, 2.036)	<.001	1.212 (1.139, 1.289)	<.001	1.220 (1.082, 1.376)	0.001	1.358 (1.213, 1.520)	<.001
In care	1.364 (0.867, 2.143)	0.179	1.256 (0.478, 3.299)	0.644	3.734 (2.190, 6.369)	<.001	0.866 (0.591, 1.268)	0.459	2.035 (1.169, 3.542)	0.012	2.688 (1.656, 4.361)	<.001
Healthcare system	1.115 (1.015, 1.225)	0.024	0.848 (0.647, 1.112)	0.233	1.158 (0.969, 1.384)	0.107	0.902 (0.840, 0.969)	0.005	0.873 (0.753, 1.012)	0.071	1.165 (1.022, 1.327)	0.022
Social housing	1.117 (1.040, 1.200)	0.002	1.162 (0.955, 1.415)	0.134	1.298 (1.136, 1.483)	<.001	1.128 (1.070, 1.190)	<.001	1.162 (1.046, 1.289)	0.005	1.282 (1.161, 1.416)	<.001
Military accommodation	1.547 (0.753, 3.179)	0.235	1.000	empty	0.437 (0.052, 3.646)	0.444	0.922 (0.482, 1.763)	0.806	1.168 (0.347, 3.925)	0.802	1.867 (0.707, 4.929)	0.208
Private sector	1.176 (1.090, 1.270)	<.001	1.035 (0.839, 1.276)	0.751	1.236 (1.072, 1.425)	0.003	1.087 (1.027, 1.150)	0.004	1.037 (0.925, 1.162)	0.534	1.204 (1.082, 1.340)	0.001
Rough sleeper	1.263 (0.619, 2.576)	0.521	1.000	empty	1.406 (0.512, 3.866)	0.509	1.461 (0.804, 2.656)	0.214	0.851 (0.291, 2.486)	0.768	1.823 (0.838, 3.964)	0.130
Refuge/shelter	1.109 (1.004, 1.226)	0.042	1.307 (1.030, 1.658)	0.028	2.084 (1.770, 2.454)	<.001	1.258 (1.162, 1.362)	<.001	1.538 (1.34, 1.761)	<.001	1.536 (1.349, 1.748)	<.001
Student accommodation	1.233 (0.760, 1.999)	0.397	0.262 (0.036, 1.914)	0.187	1.953 (1.029, 3.707)	0.041	1.380 (0.934, 2.039)	0.106	0.409 (0.127, 1.322)	0.135	0.987 (0.466, 2.092)	0.973
Relationship status												
Civil Partnership	0.648 (0.447, 0.940)	0.022	1.134 (0.494, 2.602)	0.766	0.844 (0.480, 1.483)	0.555	0.672 (0.510, 0.887)	0.005	0.404 (0.196, 0.832)	0.014	0.578 (0.341, 0.982)	0.042
Cohabiting	0.928 (0.856, 1.006)	0.071	1.094 (0.905, 1.323)	0.355	0.974 (0.859, 1.104)	0.679	1.027 (0.965, 1.093)	0.403	0.845 (0.746, 0.958)	0.008	0.952 (0.856, 1.059)	0.366
Divorced	0.902 (0.782, 1.041)	0.159	1.140 (0.807, 1.611)	0.457	1.005 (0.791, 1.276)	0.970	0.943 (0.847, 1.050)	0.286	0.947 (0.7790883 1.150612	0.583	0.782 (0.643, 0.951)	0.014
In relationship but not cohabiting	0.974 (0.896, 1.059)	0.534	0.805 (0.657, 0.986)	0.036	1.242 (1.103, 1.399)	<.001	1.045 (0.977, 1.118)	0.199	0.861 (0.756,.981)	0.024	0.971 (0.871, 1.083)	0.596
Married	0.918 (0.856, 0.985)	0.018	0.925 (0.775, 1.104)	0.389	0.733 (0.645, 0.833)	<.001	0.921 (0.873, 0.972)	0.003	0.811 (0.732, 0.898)	<.001	0.777 (0.705, 0.856)	<.001
Other	0.866 (0.685, 1.094)	0.228	1.251 (0.753, 2.080)	0.388	0.924 (0.622, 1.372)	0.694	0.855 (0.714, 1.025)	0.090	1.161 (0.855, 1.577)	0.339	0.661 (0.475, 0.921)	0.014
Separated	0.949 (0.903, 0.998)	0.041	0.905 (0.802, 1.021)	0.104	0.737 (0.674, 0.805)	<.001	0.900 (0.865, 0.938)	<.001	0.984 (0.916, 1.058)	0.665	0.924 (0.865, 0.987)	0.018
Widowed	0.804 (0.581, 1.113)	0.189	0.578 (0.142, 2.348)	0.443	0.524 (0.264, 1.041)	0.065	0.888 (0.716, 1.101)	0.278	0.989 (0.686, 1.426)	0.953	1.244 (0.897, 1.726)	0.190
Region												
East Midlands	0.708 (0.652, 0.768)	<.001	0.808 (0.664, 0.982)	0.032	0.868 (0.746, 1.010)	0.067	1.044 (0.978, 1.115)	0.197	0.797 (0.708, 0.897)	<.001	0.712 (0.638, 0.793)	<.001
East of England	1.016 (0.936, 1.104)	0.705	1.046 (0.864, 1.267)	0.642	1.310 (1.129, 1.521)	<.001	1.101 (1.029, 1.178)	0.005	0.943 (0.838, 1.062)	0.335	0.905 (0.811, 1.010)	0.076
Northeast England	0.644 (0.573, 0.725)	<.001	0.496 (0.357, 0.691)	0.000	0.770 (0.628, 0.945)	0.012	0.506 (0.460, 0.558)	<.001	0.618 (0.515, 0.742)	<.001	1.001 (0.866, 1.157)	0.992
Northwest England	0.828 (0.763, 0.900)	<.001	1.046 (0.867, 1.263)	0.638	1.206 (1.039, 1.400)	0.014	1.178 (1.103, 1.259)	<.001	0.856 (0.759, 0.964)	0.010	0.978 (0.878, 1.090)	0.688
Southeast England	1.085 (1.002, 1.174)	0.044	0.955 (0.792, 1.152)	0.630	1.127 (0.973, 1.307)	0.112	0.979 (0.917, 1.044)	0.516	0.986 (0.880, 1.104)	0.806	0.947 (0.853, 1.052)	0.311
Southwest England	0.897 (0.813, 0.990)	0.031	0.997 (0.795, 1.250)	0.978	1.205 (1.015, 1.429)	0.033	1.356 (1.253, 1.468)	<.001	1.097 (0.957, 1.258)	0.185	0.884 (0.776, 1.007)	0.064
West Midlands	0.674 (0.612, 0.741)	<.001	0.946 (0.762, 1.175)	0.615	1.344 (1.142, 1.582)	<.001	0.627 (0.581, 0.676)	<.001	0.971 (0.8502, 1.108)	0.659	0.877 (0.774, 0.994)	0.040
Yorkshire and Humberside	0.950 (0.860, 1.050)	0.318	1.167 (0.936, 1.455)	0.170	1.516 (1.280, 1.796)	<.001	0.844 (0.776, 0.918)	<.001	1.189 (1.037, 1.364)	0.013	1.111 (0.978, 1.262)	0.106
Sexuality												
Gay	1.543 (1.158, 2.055)	0.003	0.197 (0.027, 1.430)	0.108	1.525 (1.055, 2.205)	0.025	1.368 (1.092, 1.714)	0.006	1.212 (0.805, 1.824)	0.357	1.333 (0.949, 1.873)	0.097
Bisexual	1.103 (0.964, 1.262)	0.155	1.236 (0.949, 1.610)	0.117	1.934 (1.656, 2.258)	<.001	1.318 (1.178, 1.474)	<.001	1.140 (0.941, 1.382)	0.181	1.090 (0.920, 1.292)	0.320
Asexual	0.790 (0.342, 1.825)	0.582	3.192 (0.952, 10.698)	0.060	1.329 (0.529, 3.337)	0.545	0.939 (0.513, 1.717)	0.838	0.485 (0.112, 2.096)	0.333	0.759 (0.253, 2.274)	0.622
Lesbian	0.810 (0.643, 1.021)	0.074	0.421 (0.187, 0.948)	0.037	2.342 (1.817, 3.018)	<.001	1.476 (1.241, 1.756)	<.001	1.091 (0.785, 1.516)	0.602	1.182 (0.895, 1.562)	0.239
Pansexual	0.841 (0.494, 1.432)	0.524	0.937 (0.287, 3.053)	0.914	3.372 (2.083, 5.457)	<.001	1.305 (0.858, 1.984)	0.213	0.417 (0.129, 1.349)	0.144	0.349 (0.126, 0.972)	0.044
Queer	1.216 (0.499, 2.963)	0.668	1.000	empty	4.714 (2.14, 10.377)	<.001	3.162 (1.431, 6.988)	0.004	3.706 (1.353, 10.154)	0.011	1.020 (0.284, 3.669)	0.975
Ethnicity												
Asian, Asian British, Asian Welsh	0.868 (0.805, 0.935)	<.001	1.335 (1.130, 1.576)	0.001	0.896 (0.783, 1.025)	0.109	1.085 (1.024, 1.151)	0.006	1.075 (0.967, 1.194)	0.180	0.779 (0.701, 0.867)	<.001
Black, Black British, Black Welsh, Caribbean	0.859 (0.790, 0.933)	<.001	1.266 (1.057, 1.517)	0.011	0.567 (0.474, 0.677)	<.001	0.967 (0.905, 1.033)	0.317	1.053 (0.936, 1.183)	0.391	0.901 (0.806, 1.007)	0.066
Mixed or Multiple ethnic group	0.993 (0.895, 1.101)	0.889	1.054 (0.838, 1.326)	0.653	0.921 (0.778, 1.089)	0.336	0.948 (0.869, 1.033)	0.222	0.924 (0.791, 1.080)	0.323	0.980 (0.854, 1.123)	0.767
Other ethnic group	0.957 (0.834, 1.098)	0.529	1.288 (0.953, 1.740)	0.099	0.884 (0.679, 1.151)	0.360	0.987 (0.882, 1.104)	0.820	1.013 (0.833, 1.232)	0.899	0.705 (0.575, 0.864)	0.001
Gender												
Female	2.401 (2.097, 2.750)	<.001	2.739 (1.631, 4.601)	<.001	0.874 (0.728, 1.049)	0.149	0.799 (0.735, 0.869)	<.001	1.269 (1.066, 1.511)	0.007	1.043 (0.901, 1.207)	0.572
Queer, intersex, nonbinary, other	2.090 (1.019, 4.290)	0.044	1.730 (0.212, 14.135)	0.609	1.670 (0.850, 3.279)	0.136	1.004 (0.596, 1.691)	0.989	0.506 (0.107, 2.394)	0.390	0.400 (0.091, 1.754)	0.224
Age	0.992 (0.990, 0.994)	<.001	0.971 (0.966, 0.976)	<.001	0.985 (0.982, 0.989)	<.001	1.005 (1.003, 1.006)	<.001	1.019 (1.016, 1.022)	<.001	1.013 (1.010, 1.015)	<.001

## Results

### Descriptives

To take the descriptive results, almost one quarter (23.41%) of victim-survivors in the data experienced non-fatal strangulation- higher than reported in the broader literature and comparable to the proportion found in White and Majeed-Ariss’ [31] SARC sample- and 2.54% experienced abuse that resulted in harm to or loss of their unborn child (see Table 1). Over one-third disclosed feeling depressed or suicidal at the time they accessed services (41.25%), while 5.59% disclosed self-harm. Table 1 also shows that 7.33% of victim-survivors experienced injuries requiring treatment from a GP and 9.32% experienced injuries requiring A&E treatment.

Table 1 presents the descriptive statistics for the sample included in our logistic regression models. The majority of cases related to female victim survivors (96.25%), with 3.63% being cases for male victim-survivors and 0.12% ‘gender queer, non-binary, intersex and other’.^2^ The mean age was 40.56. Most victim-survivors were heterosexual (96.56%), with a small number of bisexual (1.95%), lesbian (0.77%), gay (0.48%), asexual (0.06%), pansexual (0.13%) and queer (0.05%) victim-survivors in the data. Additionally, most victim-survivors were white (78.45%) with 9.43% being for Asian and 6.64% being for Black victim-survivors. Over a third of victim-survivors lived in social housing (36.73%), a fifth lived in private sector housing (19.48%), 15.16% were in other/temporary housing and 13.51% were owner/occupiers.

Of the 77,785 victim-survivors included in the models 84.07% have experienced emotional abuse, 63.95% experienced coercive control and over half (56.57%) have experienced physical abuse. Over a third of victim-survivors disclosed experiencing financial abuse (36%), 28.25% experienced threats to kill, 20% of victim-survivors had disclosed sexual abuse and 12% tech abuse.

For needs and vulnerabilities, a minority of victim-survivors had been categorised as having support needs relating to alcohol (5.43%), drugs (4.54%) or offending (2.79%). Almost a third of victim-survivors in this sample had a disability of some kind (32.86%), 96.12% had recourse to public funds and 0.27% were pregnant. When looking at relationship status, we see that most victim-survivors are single (42.48%) or separated (24.24%). 15.45% of victim-survivors in the sample were married, 7.91% were cohabiting and 6% were in a relationship but not cohabiting. It is worth noting that relationship status is taken at point of service, meaning it may not be reflective of the relationship status at the time the abuse took place. Most victim-survivors do not currently live with the perpetrator (79.30%), but a significant minority live with the perpetrator all of the time (17.97%) or some of the time (2.73%).

### Logistic regression results

#### Factors influencing physical health outcomes

Firstly, this paper is concerned with exploring whether there are differences in physical health outcomes for victim-survivors, by experiences of abuse and by the vulnerabilities and needs of the victim-survivor (see Table 2 for full models results, including CIs). The two physical health outcomes explored in our analyses were attempted strangulation and harm to unborn baby. To take the first, those who had experienced threats to kill were four times more likely to have been strangled by the perpetrator (aOR = 4.526, *P =* < 0.001) compared to victim-survivors who hadn’t experienced this abuse type. On the other hand, those who experienced emotional abuse were less likely to have been victim of attempted strangulation than victim-survivors who hadn’t experienced this abuse type (aOR = 0.628, < 0.001). Those with offending and alcohol support needs were more likely to have been strangled than those without these needs (aOR = 1.213, *P =* 0.001 and aOR = 1.177, *P =* < 0.001). Victim-survivors of Asian or Black ethnicity were also less likely than White victim-survivors to have been strangled (aOR = 0.868, *P =* < 0.001 and aOR = 0.859, *P =* < 0.001, respectively). Gay victim-survivors were more likely than straight victim-survivors to have been strangled (aOR = 1.543, *P =* 0.003), and females were more than twice as likely than men to have suffered attempted strangulation (aOR = 2.401, *P =* < 0.001).

In terms of harm to their unborn child, in contrast to attempted strangulation, victim-survivors of Black (aOR = 1.266, *P =* 0.011) or Asian (aOR = 1.335, *P =* 0.001) ethnicity were more likely to have harm to their unborn baby than White victim-survivors. Those with a disability were more likely to have harm to their unborn baby than those without a disability (aOR = 1.107, *P =* 0.047), as were those with offending support needs compared to those without this need (aOR = 1.460, *P =* 0.002). Those living in refuge/shelter were more likely to have harm to their unborn child than homeowners (aOR = 1.307, *P =* 0.028), with no significant differences for any other accommodation types.

#### Factors associated with mental health outcomes

Mental health outcomes explored were self-harm and feeling depressed/suicidal. Victim-survivors who had experienced physical (aOR = 1.281, *P =* < 0.001), sexual (aOR = 1.907, *P =* < 0.001), emotional (aOR = 1.442, *P =* < 0.001), or financial (aOR = 1.503, *P =* < 0.001) abuse and coercive control (aOR = 1.185, *P =* < 0.001), tech abuse (aOR = 2.185, *P =* < 0.001) and threats to kill (aOR = 1.415, *P =* < 0.001) were all more likely to have self-harmed than those who had not experienced that type of abuse individually. Most of the needs/vulnerabilities included in the models increased the likelihood of self-harm (except for being pregnant and accessing by-and-for services, which were non-significant); particularly having a disability and having alcohol-related support needs, which both more than doubled the odds of self-harm (aOR = 2.179, *P =* < 0.001 and aOR = 2.297, *P =* < 0.001, respectively). Having no recourse to public funds increased the risk of having self-harmed (aOR = 1.485, *P <* 0.001, as did the perpetrator living at the victim-survivors’ address all of the time (aOR = 1,436, *P <* 0.001). In terms of accommodation, those who were in student housing (aOR = 1.953, *P =* < 0.001) or refuge (aOR = 2.084, *P =* < 0.001) were twice as likely to have self-harmed, and those who were in care (aOR = 3.734, *P =* < 0.001) almost four times as likely to have self-harmed, compared to homeowners. Those in social housing (aOR = 1.298, *P =* < 0.001) or temporary/other housing (aOR = 1.762, *P =* < 0.001) were also more likely to have self-harmed. Sexuality had a significant impact on self-harm, with gay (aOR = 1.524, *P =* < 0.025), bi-sexual (aOR = 1.934, *P =* < 0.001), lesbian (aOR = 2.342, *P =* < 0.001), pansexual (aOR = 3.372, *P =* < 0.001) and queer (aOR = 4.714, *P =* < 0.001) victim-survivors all far more likely to have self-harmed than straight victim-survivors. Black victim-survivors were over 40% less likely to half self-harmed than White victim-survivors (aOR = 0.567, *P =* < 0.001).

For the outcome of feeling depressed/suicidal, findings for associations with type of abuse were similar to those for self-harm; with experience of all abuse types significantly increasing the likelihood of feeling depressed/suicidal and tech abuse, emotional abuse and threats to kill being particularly strongly associated (aOR = 2.102, *P =* < 0.001; aOR = 1.802, *P =* < 0.001 and aOR = 1.737, *P =* < 0.001, respectively). Those with a disability were almost twice as likely to have feelings of depression/suicidality than those without a disability (aOR = 1.805, *P =* < 0.001). As was the case for self-harm, those with drug and alcohol support needs were more likely to have felt depressed/suicidal (aOR = 1.201, *P =* < 0.001 and 1.307, *P =* < 0.001, respectively). Pregnant women were slightly less likely to have felt depressed/suicidal than non-pregnant women (aOR = 0.937, *P <* 0.001); in contrast to self-harm, where pregnancy was not significant. Those accessing specialist by-and-for services were less likely to feel depressed/suicidal than those who weren’t (aOR = 0.583, *P =* 0.001), while those whose perpetrator lived at their address all of the time (but not some of the time, which was non-significant) were slightly more likely to feel depressed/suicidal (aOR = 1.112, *P <* 0.001). With regards to accommodation, those who lived in social housing (aOR = 1.128, *P =* < 0.001), temporary/other housing (aOR = 1.212, *P =* < 0.001) or in refuge/shelter (aOR = 1.258, *P =* < 0.001) were more likely to have felt depressed/suicidal than homeowners. In terms of sexuality, gay (aOR = 1.368, *P =* 0.006), bi-sexual (aOR = 1.318, *P =* < 0.001), and lesbian (aOR = 1.476, *P =* < 0.001) victim-survivors were all more likely to have felt depressed/suicidal than straight victim-survivors. Asian victim-survivors were slightly more likely to have felt depressed/suicidal than White victim-survivors (aOR = 1.085, *P =* 0.006).

#### Differences in health care help-seeking by victim-survivors

Finally, we explored whether there are differences in health care help-seeking behaviours for victim-survivors, specifically, whether they sought medical treatment from a GP or A&E following DA. The type of abuse experienced was important in terms of help-seeking behaviour; those who had experienced sexual abuse, tech abuse and threats to kill were all more likely to go to the GP (aOR = 1.224, *P =* < 0.001; aOR = 2.159, *P =* < 0.001 and aOR = 2.464, *P =* < 0.001, respectively) and A&E (aOR = 1.205, *P =* < 0.001; aOR = 2.159, *P =* < 0.001 and aOR = 2.322, *P =* < 0.001, respectively), while those who experienced coercive control or emotional abuse were less likely to visit A&E (aOR = 0.929, *P =* 0.029). Those who experienced emotional abuse were also less likely to seek treatment from the GP (aOR = 0.577, < 0.001).

Victim-survivors with drug (aOR = 1.202, *P =* 0.003 and aOR = 1.230, *P =* < 0.001), alcohol (aOR = 1.385, *P =* < 0.001 and 1.641, *P =* < 0.001) or offending (aOR = 1.437, *P =* < 0.001 and 1.497, *P =* < 0.001) support needs were significantly more likely than those without these needs to seek treatment from both a GP and A&E. Those with NRPF were significantly less likely to seek GP care than those without this additional need (aOR = 0.771, *P <* 0.001). In terms of accommodation, associations were similar to those with mental health outcomes; those living social housing, temporary/other housing or in care were all more likely to seek help from a GP (aOR = 1.162, *P =* 0.005; aOR = 2.035, *P =* < 0.001 and aOR = 1.538, *P =* < 0.001, respectively) and A&E (aOR = 1.282, *P =* < 0.001, aOR = 1.358, *P =* < 0.001 and aOR = 2.688, *P =* < 0.001), compared to homeowners. Interestingly, victim-survivors living in refuge/shelter were also more likely to seek help from both a GP (aOR = 1.538, *P =* < 0.001) and A&E (aOR = 1.536, *P =* < 0.001).

Victim-survivors who were married or in a civil partnership were less likely to seek help from either a GP (aOR = 0.811, *P =* < 0.001 and aOR = 0.404, *P =* 0.014, respectively) or A&E (aOR = 0.777, *P =* < 0.001 and aOR = 0.578, *P =* 0.042, respectively) than those who were single. Those who were in a relationship but not living together were less likely to seek help from a GP than those who were single (aOR = 0.861, *P =* 0.024), but there was no significant difference for A&E. There were no significant differences by ethnicity in visiting the GP, however, those of Asian ethnicity were significantly less likely to visit A&E than those who were White (aOR = 0.779, *P =* < 0.001). Female victim-survivors were more likely than men to seek help from their GP (aOR = 1.269, OR = 0.007), but there was no gender difference in visits to A&E.

## Discussion

This study has demonstrated that victim-survivors accessing DA specialist support services have a range of physical and mental health needs as well as need for accessing health care. While the type of abuse most associated with health outcomes varies by outcome, having additional vulnerabilities or needs was consistently associated with experiencing physical and mental health harms, as well as requiring health care from a GP or A&E.

In terms of the type of abuse experienced, the relationship with adverse mental health outcomes could not be clearer, with experience of every type of abuse significantly increasing the likelihood of having self-harmed and felt depressed/suicidal, compared to not having experienced that type of abuse individually. The strongest associations were found for tech abuse and sexual abuse, which supports previous findings on the link between these types of abuse and adverse mental health outcomes [71, 72]. This also supports qualitative insights into the experience of image-based sexual abuse (IBSA), which highlight the emotional, physical and social impact of this online victimisation and show how technology is facilitating a mutation in forms of sexual violence [66]. Those who had experienced sexual abuse, tech abuse and threats to kill were also more likely to go to the GP and A&E- however the odds ratio was lowest for sexual abuse, which could be reflective of stigma [79]. Overall, threats to kill and tech abuse appear to be particularly significant across the board in terms of increasing the likelihood of negative health outcomes. That we found positive associations between non-physical forms of DA (tech and financial abuse and threats to kill) and the occurrence of GP or A&E visits provides further support for the well-established evidence that victim-survivors visit health services for reasons other than physical injuries [80, 81].

A key finding from this study is that those who had experienced threats to kill were four times more likely to have been strangled by the perpetrator compared to victim-survivors, while those who experienced emotional abuse were less likely to have been a victim of attempted strangulation. This finding supports existing limited evidence for the link between threats to kill and NFS [75], and, taken together, these results are indicative of the tipping point from emotional abuse to threats to kill which then escalates to attempted strangulation. These results should also be considered within the broader context of coercive controlling behaviour, a systematic pattern of behaviour that establishes dominance over another person, including through threats of violence [82]. It is crucial to intervene before this point of escalation, given that threats to kill are a risk factor for intimate partner homicide (IPH) [73, 76] and domestic homicide [74]. The meta-analysis for risk factors of IPH [73] found that the probability of IPH increases 11.36 times with a death threat, 6.7 times with a previous strangulation attempt, 5.83 times in the presence of controlling behaviours and 3.74 times if the victim-survivor is abused during pregnancy. In contrast to this and other studies (e.g. [30, 83]), the current study found no significant relationship between being pregnant and having experienced attempted strangulation. This may be due to the way pregnancy is recorded, whereby pregnancy at the time of accessing the service is measured, as opposed to being pregnant at the time of the abuse.

Also pertinent is the finding that victim-survivors accessing specialist by-and-for services are less likely to feel depressed/suicidal, although this finding needs to be considered parsimoniously due to the relatively low number of by-and-for services present in our data. Our findings echo existing work which has highlighted the complex needs of women accessing specialist DA services [53] and the value of by-and-for services for Black and minoritised women experiencing DA and mental ill-health [84, 85]. This finding should also be interpreted in the context of a recent qualitative systematic review of help-seeking for DA among ethnic minority women in the UK, which found that many women could not disclose to or seek help from statutory services, access refuges, receive benefit, or have access to appropriate housing as they had no recourse to public funds due to their immigration status [86]. Staff at mainstream services denied and rationalised the existence of DA against BME women, which was a barrier to further disclosure or desire to seek help. Therefore, there is a lot we still don’t know about the needs and experiences of this population of women.

The type of accommodation was strongly associated with victim-survivors health outcomes in the current study. Those living in social housing, temporary/other housing, refuge/shelter or in care were all more likely to seek help from a GP and A&E than homeowners. Victims-survivors living in refuge/shelter were also more likely to have experienced both a perpetrator causing harm to their unborn child and attempted strangulation. Together these findings suggest those seeking refuge may have been in severely abusive relationships with high risk to themselves and their baby, which could have catalysed fleeing their home and seeking help from health care professionals to keep their baby safe [87]. This finding could also reflect the fact that victim-survivors in refuge/shelter receive support with accessing health care services. Or, the other way around, that approaches like the aforementioned IRIS(+) programme are increasing referrals made to specialist support services by GPs and other health professionals [56, 88]. Qualitative research with victim-survivors has found that clinicians being insufficiently trained and supported to engage is a barrier to disclosing DA in healthcare settings, which can be compounded by internal barriers such as shame and self-blame, but that over time GP-led disclosures can inspire feelings of hope and engagement with specialist DA advocates [89]. Temporal analysis would help to better understand victim-survivors’ help-seeking journeys through services.

Those who were in student housing or refuge/shelter were twice as likely to self-harm as homeowners, while those who were in care (children’s home/foster care) were almost four times as likely to self-harm. Those in social housing or temporary/other housing were also more likely to have self-harmed. The significance of being in student housing and in care, combined with the strong links found between tech and sexual abuse and self-harm, is in line with existing findings on the high prevalence of these types of abuse in adolescence and/or school/college settings and links with adverse mental health [72, 90–92]. Particularly PTSD, which is common amongst young women with experience of DA and often comorbid with depression [49, 93]. It could also reflect a lack of support for and culture conducive to these types of harmful behaviours in schools and universities [94, 95], and/or the struggle experienced by the specialist support sector to keep up with the changing dynamics of tech abuse [96].

Reflecting the association between accommodation status and help-seeking behaviour and self-harm, those who lived in social housing, temporary/other housing or in refuge/shelter were more likely to have felt depressed/suicidal than homeowners. Together these findings highlight the intersection between DA and housing instability/homelessness and poor health outcomes [97–99]. Interestingly, being in student housing was not associated with feelings of depression/suicidality but although it was with self-harm, suggesting students are at risk of self-harm despite being less likely to report that they feel depressed/suicidal. As alluded to above, this could have more to do with disclosure reluctance than actual lower levels of depression/suicidality, although it is interesting that students might be more willing to disclose self-harm than feeling depressed/suicidal. The (mental) health outcomes and help-seeking behaviours of students with experience of DA should be explored further, as it may be the first time they are living independently so they may have less access to their ‘usual community’ as a protective factor.

Those with a disability were around twice as likely to have self-harmed and felt depressed/suicidal, and were also more likely to have harm to their unborn baby and to have required a visit to A&E. This is in line with findings from the systematic review by Breckenridge et al. [61], and could be because barriers to care often faced by women with physical and mental disabilities may be overridden by immediate treatments needs which catalyse health service use, especially for those who are pregnant who may seek health care help out of concern for their baby’s health over their own health and wellbeing.

Our study highlighted some important differences with regards to ethnicity. Black and Asian victim-survivors were more likely to have experienced abuse causing harm to their unborn child than White victim-survivors, and Asian victim-survivors were slightly more likely to have felt depressed/suicidal but significantly less likely to visit A&E. The aforementioned systematic review highlighted the importance of family honour and identity among the South Asian community, meaning disclosure and help-seeking can be likened to dishonouring their community and cause women to feel a sense of shame, denial, loss of identity and lack of choice which prohibits them from seeking help [86]. On the other hand, evidence suggests that victim-survivors from the British South Asian community do access and engage with specialist non-statutory services [100–102], highlighting the importance of these types of services. Victim-survivors of Asian or Black ethnicity were also less likely than White victim-survivors to have been strangled, which might suggest that strangulation within the context of DA is a problem predominantly experienced by white people. However, a previous quantitative analysis of the determinants of NFS found that ethnicity was not significantly related to the frequency of NFS [75], and a recent meta-analysis of factors associated with NFS found that identifying as a Black woman was significantly related to NFS victimization [103].

It is important to bear in mind that the current analysis was based on a sample of victim-survivors who have successfully accessed support and some marginalised groups, including black and ethnic minoritised women, those with insecure migration status and those with NRPF, are less likely to be able to access support and/or less likely to disclose DA. Furthermore, intersecting inequalities compound barriers to disclosure and help-seeking [104–107]. Therefore, any findings in relation to ethnicity should be considered in light of structural inequalities and their effect on barriers to accessing services [85, 108]. Further research based on samples with larger minority ethnic groups, including those not engaging with specialist support services, would help to better understand the relationship between ethnicity and NFS. Future research should also look at the ethnicity of the perpetrators of NFS, as this may be more meaningful to understand the relationship between patterns of behaviour and ethnicity.

### Strengths and limitations

The current study has two considerable strengths. Firstly, that our analysis is based on a large national dataset from a membership/federation specialist DA service. This extends the existing evidence base on health outcomes and health care utilisation of victim-survivors of DA, within which most studies are small samples from clinical trials or small observational studies. Secondly, this study was co-produced with stakeholders from Women’s Aid, benefitting from their input from the point of developing research questions to the interpretation of results.

With regards to limitations, we did not gather insights from victim-survivors, which would have enhanced the depth of perspective. Furthermore, while our study is looking at victim-survivors’ experiences, we acknowledge that the source of the abuse is the perpetrator’s behaviour, and to gain a deeper understanding and insight analyses should look at perpetrator characteristics. Women’s Aid have already improved the data capture by On Track so that the type of abuse experienced relates directly to the perpetrator, and changed the language used to place the responsibility for the behaviour firmly on the abuser (e.g. “Has the perpetrator ever used…”). Secondly, the data analysed pertains to a population who had successfully accessed DA services, which means the characteristics and experiences of those not disclosing DA and not in receipt of support- who we know are likely to be those with the highest need [104–106]- are not considered. Another key limitation of this study is the exclusion of approximately 55% of cases due to missing data across key variables. We acknowledge that this reduces generalisability and may have excluded individuals with more complex types of abuse and vulnerabilities. However, the missing data were not missing at random, and individuals with incomplete records had different health and health service outcomes. Given this, we chose not to use any form of imputation, as doing so would have required us to assume that those lost due to data missingness had similar profiles and outcomes to those retained. This decision prioritised internal validity and interpretability, while recognising the trade-off in representativeness.

Finally, as has already been discussed as a common limitation of studies in this area, it is not possible from our analysis to establish causal links between abuse, needs and vulnerabilities and physical and mental health outcomes- particularly regarding the latter, one could be influencing the other or vice versa, or the relationship could be working in both directions. Findings from the qualitative literature suggests the direction of the relationship, for example describing a ‘turning point’ where realising the impact of the abuse motivates women to change their situation [109, 110], but quantitative findings are primarily associations, and few studies exclude those with mental health issues at baseline. While there is scientific value in better understanding the relationship between DA and mental health, there are also moral/ethical implications of doing this, because it means the most affected people and those who need help the most end up excluded from studies. Furthermore, even for those with mental health issues that pre-dated an abusive relationship, experiencing DA can exacerbate existing issues, although most studies do not measure clinical thresholds. Regardless, the current study has contributed to the ever-growing literature which supports the negative impact of DA on victim-survivors’ mental health.

### Implications

Our findings carry several important implications. Firstly, it is crucial that health care providers better recognise the signs of and risks associated with coercive control and emotional abuse, which our findings show are more strongly associated with victim-survivors’ mental health than physical abuse; and coercive control also increases the risk of being strangled. This finding supports the conceptualisation of NFS as part of an ongoing and escalating pattern of coercive control [27, 75], which, with the right understanding and training, health care providers have an opportunity to spot and intervene before someone is strangled. Our findings also suggest that those experiencing emotional abuse and coercive control are less likely to visit either a GP or A&E, demonstrating the need for other health care professionals and professionals in other services to be able to recognise and refer victim-survivors of these types of abuse. Similarly threats to kill, whilst non-physical themselves, increased the risk of NFS four-fold in the current study, and are also a risk factor for domestic and intimate partner homicide, and thus must be taken seriously and dealt with before the threat becomes a fatality. As part of this, health care professionals and other agencies involved in the DA response need to be aware of the signs (including non-visible) and risk of strangulation.

Research–practitioner collaborations are critical for improving the response to non-fatal strangulation in DA cases [111], including police, health care professionals and specialist DA support services. Combined expertise and cross-training between these agencies has the potential to increase visible detection and documentation of signs of strangulation, screening for non-visible symptoms, referral of victim-survivors to support services who can provide resources and support and make necessary onward referrals where more specialist expertise is required. Input from researchers can provide the necessary context for this work and ensure new approaches avoid reinforcing misconceptions about strangulation. Treatment services for perpetrators of NFS and interventions to support and empower victim-survivors to leave perpetrators of coercive control are a key part of this [75]. And finally with regards to training in health care settings, there is a need for training for clinicians around risks of tech abuse. With technology-facilitated forms of abuse on the rise, experts in this field have identified that digital harms are not captured by existing safeguarding tools and there is a lack of guidance for health practitioners on how to address these risks [112]. It is recommended that hospital guidance and safeguarding training needs a digital update to ensure clinicians can effectively care for vulnerable patient groups, particularly those who appear to be more at risk, including young women and girls, LGBTQ youth and religious and ethnic minorities.

Our findings also highlight the need for increased funding and availability of specialist by-and-for DA services, which are more likely to meet the mental health needs of minoritised victim-survivors- including interventions specifically designed to support migrant victim-survivors [113], although we acknowledge the number of by-and-for included in our analyses was relatively small. As well as more focus on the cultural sensitivity of services, our findings indicate a need for services specifically tailored towards the needs of victim-survivors with socioeconomic vulnerabilities such those in insecure housing/unstable accommodation, who appear to be at heightened risk. There is a need for greater socioeconomic security for victim-survivors of DA, including safe and stable housing. Consideration must also be given to those in student housing, who our study found to be at greater risk of adverse mental health outcomes but less likely to seek health care help. Alongside much-needed specialist service provision, statutory services must also be culturally appropriate and trauma-informed to be able to meet the needs of specific groups. Polices must be directed at protecting survivors of DA, funding streams must be put in place to address the long-term social and economic needs of victim-survivors, and interventions should focus on women's autonomy and agency and provide flexible long-term services that are tailored to women's needs. While we are seeing positive movements in these areas, there is still a need for deeper understanding of the intersection between DA, housing instability/homelessness and health, and how to apply this knowledge toward the development of more holistic, coordinated and effective responses to these (and other) co-occurring problems [53, 97].

An implication for researchers is that we should be trying to use more robust causation models such as randomised-controlled trials and structural equation modelling, where ethically viable. The majority of the quantitative evidence base is studies of associations, which cannot establish causality, and most studies do not measure the clinical scale of mental health. Where it is not morally, ethically and practically possible to employ such study designs, large scale mixed methods studies should be conducted, such that quantitative findings can be contextualised using qualitative data. Building lived experience perspectives into research from start to finish is also critical for gaining a fuller understanding of experiences of DA and its impact upon health.

## Conclusion

This study has drawn upon often underutilised administrative data collected by domestic abuse services to demonstrate the devastating outcomes of experiencing many different types of DA, and having additional needs/vulnerabilities, on victim-survivors physical and mental health, and health care help-seeking behaviour. Critically, our findings highlight the almost inevitable harms to mental health for victim-survivors of DA, the dangers of non-physical types of abuse such as threats to kill and tech abuse and the heightened risk of attempted strangulation. Health professionals, specialist support workers, researchers and policymakers must continue to explore more joined-up ways of working to further improve the response to DA and intervene before irreversible damage is done. Given that suicide has been identified as the most common cause of death for victim-survivors of DA for the second year running [114], the need has never been greater.

## Data Availability

The data used in this paper is subject to a legal data sharing agreement between Women’s Aid Federation of England (WAFE) and City St. George’s, University of London. Data may be available from WAFE subject to further data sharing arrangements.

## Abbreviations

DA: Domestic abuse
IPV: Intimate partner violence
DV: Domestic violence
DVA: Domestic violence and abuse
NFS: Non-fatal strangulation
PTSD: Posttraumatic stress disorder
GP: General practitioner
A&E: Accident and emergency
CYP: Children and young people
IPH: Intimate partner homicide

## Footnotes

1 It is important to acknowledge that harm to or loss of unborn child is attached to the perpetrator’s behaviour, however for brevity in places such as tables and figures we sometimes refer to ‘harm to or loss of unborn child’ or ‘harm to unborn child’.

2 While we acknowledge that this slightly conflates sex and gender, we are reporting as the data is presented on On Track.
